# A hierarchical, compact and efficient phenanthrene supramolecular polymer light harvesting antenna

**DOI:** 10.1039/d6tc00644b

**Published:** 2026-06-11

**Authors:** Romain Brisse, Ioan Iacovache, Jovana Jevric, Simon M. Langenegger, Benoît Zuber, Robert Häner

**Affiliations:** a Department of Chemistry, Biochemistry, Pharmaceutical Sciences, University of Bern Freiestrasse 3 CH-3012 Bern Switzerland romain-jh.brisse@protonmail.com; b Institute of Anatomy, University of Bern Baltzerstrasse 2 CH-3012 Bern Switzerland

## Abstract

Reaching extremely high levels of sophistication of naturally occurring supramolecular polymers (SPs) with artificial structures represents a paradigm with important underlying application potential in various fields such as biomaterials and optoelectronics. A key challenge in synthetic SP research is mimicking the complex hierarchical and multiple level folding of natural SPs, such as DNA for instance. In previous works, we have developed an artificial mimic of the chlorosome pigment antenna, consisting of micrometer-long phenanthrene-based SP nanofibers, with exceptional light-harvesting properties. In the present work, we have advanced this system one step further, by assembling the Trimer A nanofibers into sophisticated hierarchical nanostructures. To our knowledge, this work is the first report of hierarchical nanostructures of a functional synthetic SP. The polymerization was monitored by means of UV/visible and fluorescence spectroscopy. Cryo-EM and AFM revealed high aspect-ratio nanoribbons as well as large annular nanostructures. Our thorough study with acridine orange as the energy acceptor shows that the excellent light-harvesting antenna effect is preserved. The annular nanostructures observed are unprecedented in the field of synthetic hierarchical SPs, and this constitutes a notable chemical achievement. We also report the first calculation of a dimensionality compression factor, and we found a remarkable two-orders-of-magnitude reduction for an annular structure. Overall, the present work shows that dimensional compression does not impede light-harvesting performance. It paves the way, in SP science, for potential applications such as advanced energy materials.

## Abstract 2

Unprecedented hierarchical nanostructures of functional, light-harvesting supramolecular nanofibers are reported. An efficient antenna effect is maintained.

## Graphical abstract

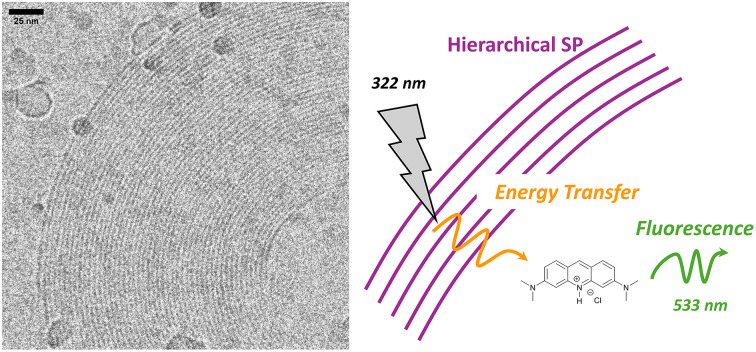

## Introduction

**Fig. 1:**
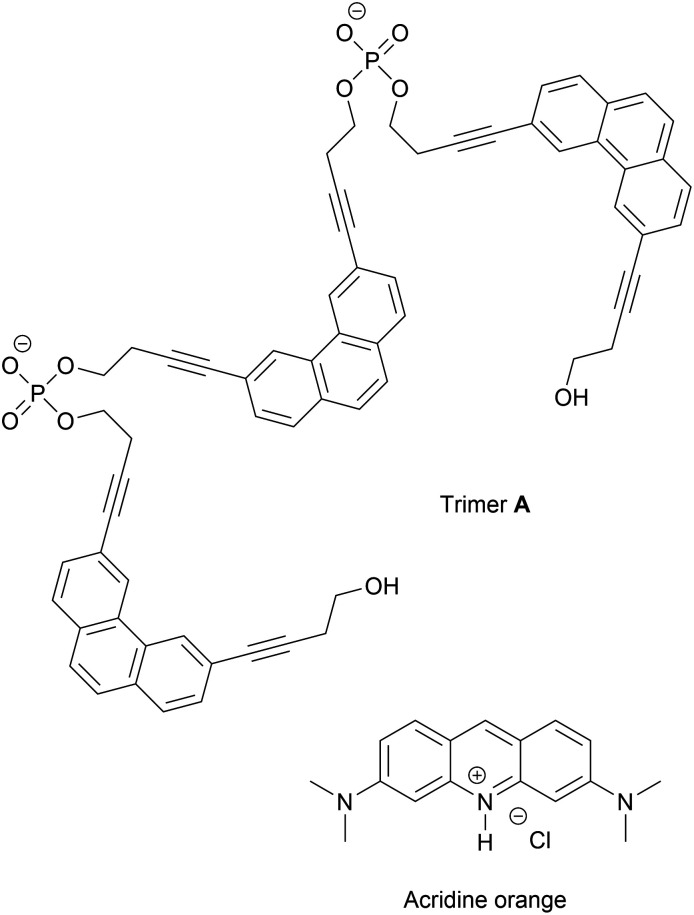
Chemical structures of Trimer A and acridine orange.

Arising from the supramolecular nature of their constitutive interactions,^1^ supramolecular polymers (SPs) offer new potential to address current bottlenecks in materials sciences. SPs present specific and promising properties including for example, stimuli responsiveness, ease of recyclability and processability or self-healing.^2–8^ Research on SPs has already led to the development of promising materials with several original functionalities, especially in the field of biomaterials or (opto-) electronics.^9–15^ In our work, we are developing functional SPs with unique light-harvesting antenna capabilities,^16–19^ building on small amphiphilic synthetic molecules and closely mimicking a chlorosome, one of the most efficient natural photosynthetic antenna complexes.^20^ In particular, we have shown how an organic 3,6-dibutynylphenanthrene phosphodiester-linked trimer (called Trimer A in the present work, see Fig. 1) can self-assemble into 1D nanofibers with exceptional light-harvesting properties, in an aqueous environment.^16^

In naturally occurring SPs, the polymer is ubiquitously engaged in highly complex organizations,^21–24^ which the synthetic structures developed up to now are far from being able to reach. One of the most critical features of many natural SPs is their hierarchical organization that, for example, provides them with a compact dimensionality, to fit in tiny cellular spaces, such as organelles. For instance, the successive multi-level hierarchical folding of the DNA double helix into chromosomes, by histone proteins, provides a remarkably compact material with a dramatically reduced spatial footprint, which fits the size of a nucleus.^25^ Hierarchical and compact arrangements consisting of lamellar structures of chlorophyll stacks are, besides, also essential to natural chlorosomes themselves, to ensure an extraordinarily high and functional concentration of pigment within the membrane of the light harvesting organelle.^20^ Being able to engage a given synthetic SP into higher order, hierarchical nanostructures is an important challenge in current research in the field.^2,26,27^ Finding a window of experimental conditions where a given SP maintains its primary structure, while also involving it in further levels of organization is a complex and still poorly addressed scientific question. This is because processes and rationales governing SP stability, are still far from being well understood and the science in the field is still in its infancy. Noteworthily, within the few reported artificial structures where a SP is engaged in a higher order supramolecular assembly, the nanofiber has often formed the first level of association. Yagai *et al.* have shown how to induce an additional level of internal ordering within a pre-existing nanofiber, providing nano- and helical coils.^27,28^ Other groups have also shown hierarchical SP organizations, with the association of several nanofibers within flat ribbons, 2D-nano arrays or super-helices.^29–31^ The present work comes within these pioneering studies. We discovered a set of conditions that resulted in the formation of hierarchical nanostructures of Trimer A nanofibers. In comparison with the existing literature, this work constitutes, to our knowledge, the first example of the hierarchical self-assembly of a functional synthetic SP. The supramolecular polymerization was characterized in detail by means of UV/visible and fluorescence spectroscopy. Our thorough cryo-EM and AFM studies evidenced higher order nanoribbons of Trimer A nanofibers and also, very surprisingly, unprecedented and annular structures. We performed a comprehensive assessment of the impact of such a hierarchical arrangement on the antenna properties of the system. We resorted to acridine orange (AO) (see Fig. 1) as a probing dye to play the role of the energy sink. We observed that the strong light-harvesting antenna properties are maintained. It is also noteworthy that within the newly developed annular structures, a significant level of compression for a hierarchical SP assembly was reached and we have been able to quantify the effect by a compression factor calculation.

## Results and discussion

### Formation of hierarchical and compact structures of Trimer A supramolecular polymer nanofibers

**Fig. 2:**
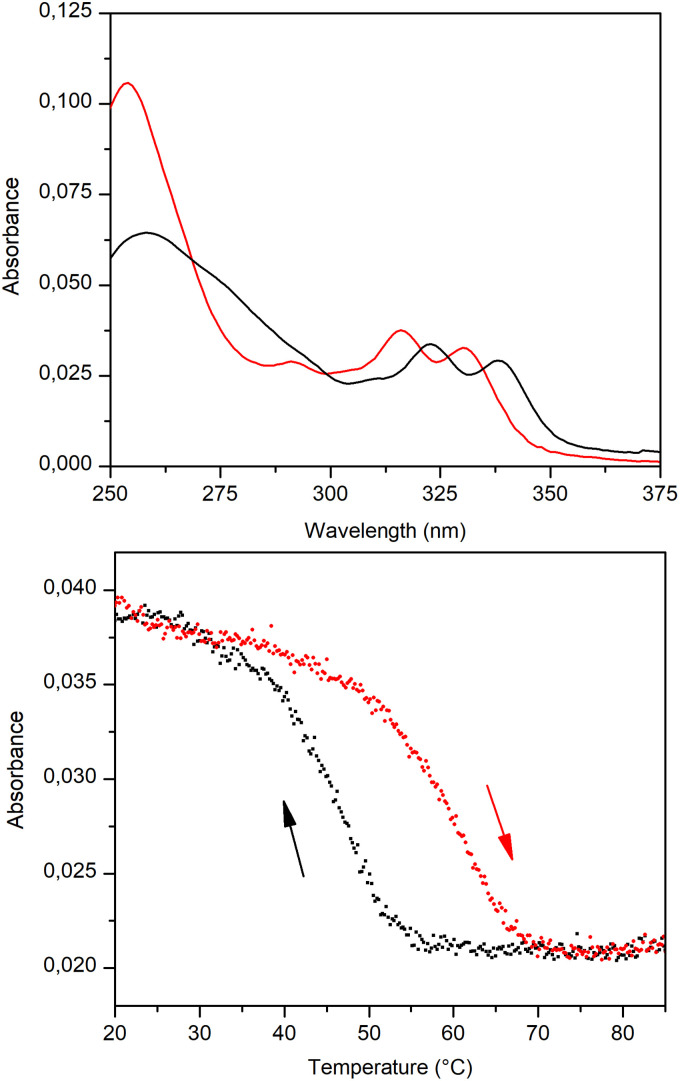
Top: UV-visible spectra of the disassembled Trimer A (85 °C, red) and the assembled polymer (20 °C, black). Bottom: Temperature dependent Trimer A absorbance at 342 nm, during the 1 °C min^−1^ cooling ramp (black) and when a subsequent additional 1 °C min^−1^ heating ramp was applied, after the cuvette had reached 20 °C (red). Conditions: TEOA (0.12 M), HCl (85 mM), Trimer A (0.5 µM); pH 7.4.

The synthesis of Trimer A was performed according to a previously reported procedure.^16^ The supramolecular polymerization reaction was initiated by applying a temperature gradient of 1 °C min^−1^ to an aqueous solution of Trimer A buffered (pH 7.4 at 25 °C) with triethanolamine (TEOA), starting from 85 °C, down to 20 °C (*cf.* SI). To characterize the effect of SP formation, UV/visible spectra were collected at 85 °C and at 20 °C (see Fig. 2). For the two lowest energy absorption bands of phenanthrene at 316 nm and 331 nm, we observed a redshift of 7 nm when cooling down, associated with a slight hypochromicity. In the higher energy region, we observed that the band at 254 nm shrinks in intensity while widening and slightly red shifting (4 nm) upon cooling. In the course of the supramolecular polymerization reaction, the absorbance of the phenanthrene was also followed at 342 nm (*cf.*Fig. 2). In agreement with the UV/visible spectra, we observed an enhancement of the absorption upon cooling, with a transition starting at *ca.* 55 °C, completing at *ca.* 30 °C and being centered at 45 °C. We also investigated the heating curve of the assembled structures. A transition was observed, starting at *ca.* 35 °C, being complete at *ca.* 70 °C, with a center at 57 °C. We also observed a strong hysteresis between the heating and cooling curves (*Δ* = 12 °C). Hysteresis is a complex kinetic phenomenon that often occurs in supramolecular polymerization processes. For the present system, hysteresis is in the same range as that observed for non-hierarchically arranged Trimer A nanofibers.^16^ Its origin can be multiple, for example, the polymerization process could be less favorable than the depolymerization process. This asymmetry could find its origins in the presence of kinetic barriers, such as nucleation barriers.^1^

Temperature dependent steady state fluorescence spectra were also acquired, and the results are presented in Fig. S1. Alongside a strong hypochromic shift and widening of the fluorescence band, we observed a strong red-shift of the fluorescence maximum, from 393 nm to 431 nm upon cooling from 85 °C to 20 °C. UV/visible and fluorescence spectroscopy results presented here are in line with our previous studies and indicate that the supramolecular polymerization of Trimer A can also be done in a TEOA buffer.

**Fig. 3:**
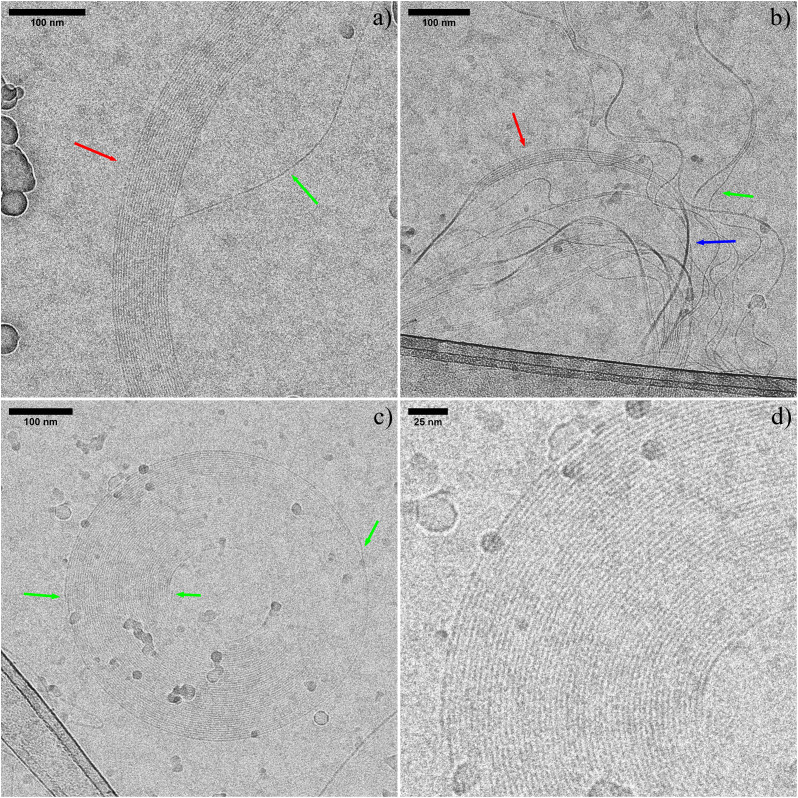
Cryo-EM pictures of Trimer A solution after self-assembly, showing the morphologies observed. (a) and (b) green arrow: individual single fiber; red arrow: nanoribbon observed face-on; blue arrow: nanoribbon observed edge-on; (c) green arrow: single fiber tail of the nanostructure; (d) zoomed in portion of an annular structure, showing the hierarchical arrangement of 1.7 ± 0.5 nm nanofibers. Conditions: TEOA (0.12 M), HCl (85 mM), Trimer A (0.5 µM); pH 7.4.

Cryogenic electron microscopy (cryo-EM) enabled us to characterize the morphology of the SP. Details of experimental sample preparation conditions can be found in the SI. Thorough investigations of the cryo-EM sample indicated well-defined nanostructures, as exemplified in Fig. 3 and the SI (Fig. S2–S4). Micrometer long nanofibers with 2.3 ± 0.4 nm width (Fig. S5) were identified, in agreement with the approximate dimensions of two Trimer A molecules interdigitating with each other, with face-to-face arrangement of π-stacked phenanthrenes and, hence, in line with our previous works.^16,19^ Surprisingly, we also discovered that the nanofibers associate together to form high aspect ratio micrometer long nanoribbons, providing the system with a second order of organization (Fig. 3). The ribbons were oriented either in a face-on or in an edge-on perspective. Face-on observation reveals they are composed of 2 up to *ca.* 20 parallel aligned nanofibers. We measured a width of 1.8 ± 0.4 nm for a single constitutive nanofiber (Fig. S6). This dimension is slightly smaller than that for a non-associated single nanofiber. We believe that the association of the nanofibers induces slight structural modifications of the interdigitating supramolecular arrangement of Trimer A. As can be expected, ribbons observed from the edge-on point of view, appeared with a higher contrast. We measured an average width of 4.1 ± 0.6 nm, independent of the number of associated fibers (Fig. S7). We assume that in such a configuration, a ribbon is not perfectly perpendicular to the TEM grid, giving an apparent width higher than the one of individual nanofibers. Interestingly, we also observed structures with an annular morphology (Fig. 3(c), (d) and Fig. S3, S4). The structure represented in Fig. 3(c) and (d), is of a circular shape, for which we measured an outer diameter of *ca.* 470 nm and an inner diameter of *ca.* 150 nm. The sub-structure was also very well resolved as shown in Fig. 3(d) and revealed again a hierarchical organization. We could distinguish an impressive concentric annular arrangement of *ca.* 50 layers of material. We measured a size of 1.7 ± 0.5 nm for each layer (Fig. S8). This value is in the same range as the one observed for the fibers forming the ribbons. Thus, the structure can be regarded as a circularly arranged nanoribbon. It is also not clear how many fibers the structure is made of but the presence of 2 tails situated at the outer edge could indicate a spiral organization, with at least two individual fibers. A similar annular structure is observed in the cryo-EM image displayed in Fig. S3. It reveals a structure partially folded on its outer edges, which suggests a certain level of mechanical flexibility. Eventually, in some cases, an additional third level of organization was observed. We eventually noted that the further association of ribbons into a thicker ribbon morphology, when in the edge-on orientation, is also possible (Fig. S4). This confirms the propensity of the supramolecular nanofibers of phenanthrene to adopt hierarchical structuration in the present experimental conditions.

These results represent to our knowledge the first evidence of a functional (light-harvesting) synthetic SP arranging into higher order organizations. Importantly, the experimental conditions presented here can also be viewed as a means of compacting the nanofibers and reducing the overall SP dimensionality. This is especially true when considering the circularly arranged nanoribbon described above. For this structure, we calculated that approximately 50 µm of nanofiber material was packed into a space with a characteristic dimension of about 500 nm. This corresponds to a two orders of magnitude compression factor. This represents a unique and significant new feature for the 1D phenanthrene SP nanofibers, which is a direct consequence of the hierarchical assembly. To our knowledge, we are also the first to calculate such a compression factor for a hierarchically ordered artificial SP. This remarkable result is reminiscent of the DNA compression in cell nuclei (for which the compression factor reaches impressive 5 to 6 orders of magnitude values, between the native double helix and the chromosomic highly compact state).^32^

**Fig. 4:**
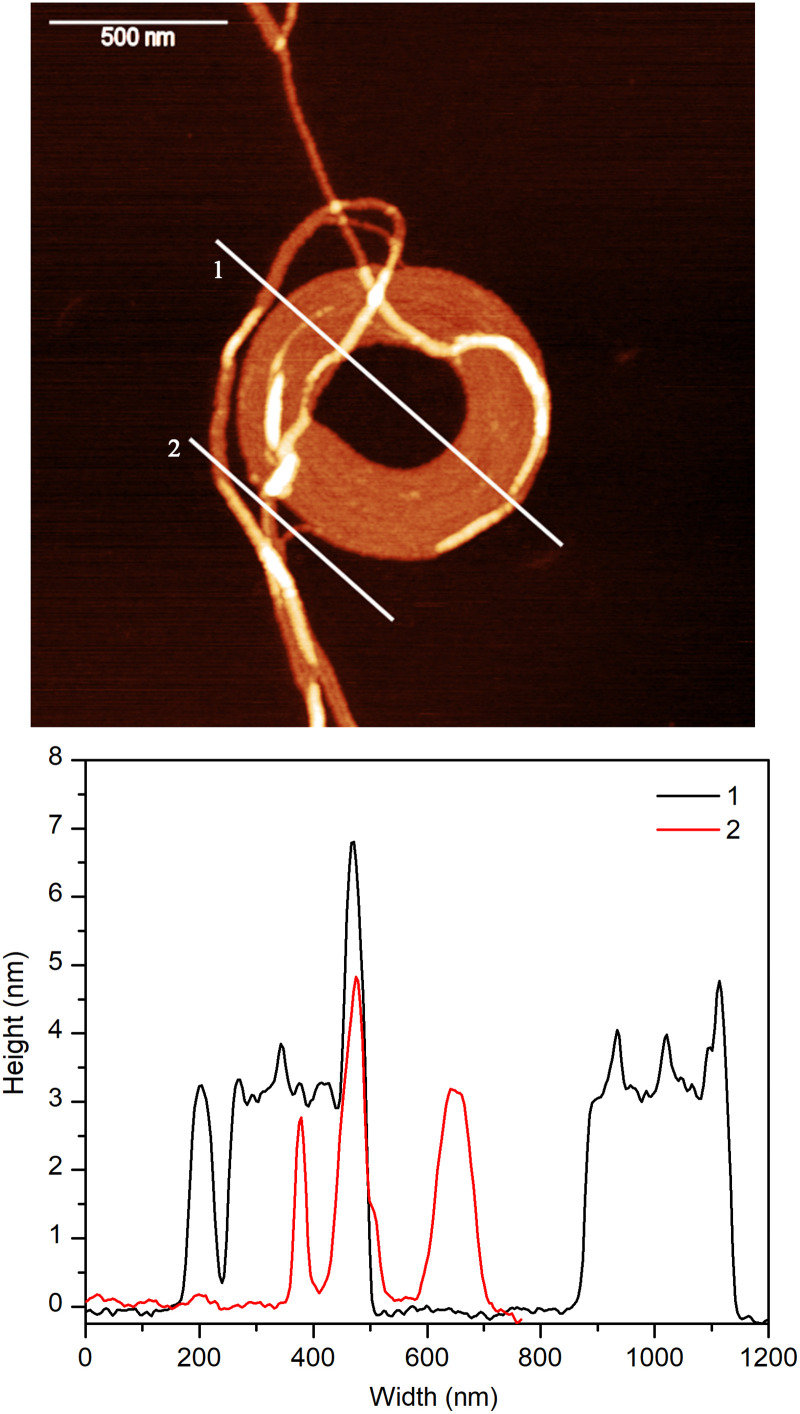
An AFM picture of Trimer A after self-assembly, showing the morphology observed, with the corresponding height profiles. Conditions: TEOA (0.12 M), HCl (85 mM), Trimer A (0.5 µM); pH 7.4. APTES-modified mica.

Furthermore, we also characterized the structures using atomic force microscopy (AFM). The samples were deposited onto APTES-modified mica (*cf.* SI for more details regarding the sample preparation). In agreement with the cryo-EM results, we were able to observe micrometer long nanofibers, the smallest apparent height that we measured was 0.3 nm (*cf.* Fig. S9), which we attribute to a single nanofiber. This value is smaller than the one measured with cryo-EM (2.2 nm), but is in the range of previous results, for similar linear SPs.^16,33^ It is well known that the heights measured by AFM are underestimated, due to tip–sample interactions, with our structures.^33^ Filament-like bundles of fibers were also detected, as shown for instance in Fig. S9. An apparent height up to *ca.* 2.2 nm was measured for the thickest bundle presented in Fig. S9. Considering a size underestimation factor identical to that observed for single nanofibers, we conclude that this thick filament-like object is in fact a bundle of 2.2/0.3 ≃ 7 nanofibers. We propose that such a bundle could be a nanoribbon (such as the ones observed by cryo-EM), composed of 7 nanofibers and lying in the edge-on position on the APTES-modified mica-sample. In Fig. 4, a *ca.* 500 nm wide annular structure with an apparent height of 3 nm is seen. Fig. 4 also indicates that this structure results from the circular coiling of filament-like bundles, as evidenced by the long tails emerging from the annulus and the visible circular stripes within the structure. Again, considering a size underestimation factor identical to the one observed for a single nanofiber, the annular structure observed on AFM has a thickness of 3/0.3 = 10 nanofibers. This differs strikingly from the cryo-EM detected structures, the thickness of which corresponds to 1 nanofiber (*ca.* 1.8 nm). Therefore, the annular objects observed by AFM could be 10 times thicker. In other words, we detected different annular objects with AFM as compared with cryo-EM. This discrepancy illustrates the inherent limitations of the AFM technique at the solid–air interface. In this case, the sample preparation includes a deposition/drying phase on the APTES-modified mica where a state of further aggregation of the nanostructures could be captured or a reorganization occurs. Cryo-EM represents structures that are closer to the native state in solution.

The present work sheds light on the discovery of a set of conditions leading to the formation of hierarchical and compact nanostructures of Trimer A nanofibers. This result constitutes in itself a noticeable chemical achievement. Further comprehensive studies to elucidate the mechanisms behind will be needed. The polymerization is indeed operated in a multiparametric framework, which comprises a complex aqueous environment consisting of large excesses of TEOA, TEOA–H^+^ and Cl^−^. In comparison to our previous studies with Trimer A in a sodium phosphate buffer, the full ionic environment is thus novel here. Whether this environment has an impact on the hierarchical nanostructure formation represents a vast scientific question that the present work opens.

We have presented the assembly of phenanthrene nanofibers into hierarchical and more compact nanoribbon structures.

### Study of the antenna effect with acridine orange as the energy acceptor

**Fig. 5:**
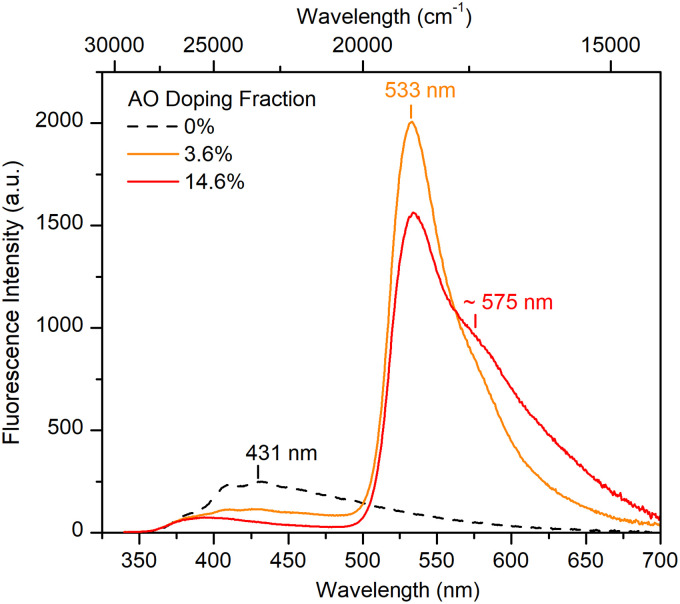
Fluorescence emission of assembled Trimer A, without (dashed line) and in the presence of two different doping fractions of AO: 3.6%, (orange) and 14.6% (red), at 20 °C. Conditions: TEOA (0.12 M), HCl (85 mM), Trimer A (0.5 µM); pH 7.4; *λ*_ex_: 322 nm.

We further investigated whether the light-harvesting antenna functionality previously observed for phenanthrene fiber SPs was also preserved by the present architectures. With a strong fluorescence usually centered at *ca.* 530 nm in an aqueous environment and a strong absorption at *ca.* 490 nm, overlapping the fluorescence of the phenanthrene SP (*cf.* Fig. S1), acridine orange (AO) constituted a suitable probe to assess the energy transfer capabilities of the synthesized hierarchical SP.^34^ To do so, we added gradual doping fractions of AO from 0.1% up to 14.6% to the SP (doping fractions refer to the molar ratio of AO per unit of phenanthrene). The fluorescence emission spectra were collected, after selective excitation of the phenanthrene chromophore at 322 nm (Fig. S11). An estimation of the corresponding fluorescence quantum yield (*Φ*_F_) was also calculated. All the spectra, *Φ*_F_ calculation data and plots as a function of the doping fraction can be found in the SI (Table S1 and Fig. S17). A selection of emission spectra at key doping fractions is presented in Fig. 5. Alongside the doping experiment we observed the concomitant decrease of the phenanthrene fluorescence maximum at 431 nm and the soaring of a fluorescence band at 533 nm, corresponding to the fluorescence of AO. The band reached its maximum intensity at a doping fraction of 3.6%. This was also in line with a sharp increase in the system fluorescence quantum yield, from *Φ*_F_ = 3.1% up to *Φ*_F_ = 9.1%. At higher fractions of AO, we observed a slight decrease in the intensity of the fluorescence band at 533 nm and the appearance of a new band, centered at *ca.* 575 nm. Overall, the quantum yield remained roughly stable (*Φ*_F_ = 8.7% at a doping fraction of 14.6%). AFM also confirmed the stability of the SP after the doping experiment (Fig. S10). The excitation spectra of the system, probed at 575 nm for each doping fraction revealed that the AO fluorescence always originated from the phenanthrene SP excitation, with a continuously growing phenanthrene contribution (*cf.* Fig. S12). Besides, in the absence of SP and in otherwise identical conditions, the fluorescence emission of AO could hardly be detected (*cf.* Fig. S14). These two results attributed undoubtedly the strong fluorescence of AO, detected during the doping experiments, to energy transfer from the phenanthrene SP to AO. The overall decrease of the phenanthrene donor emission band is concomitant with the rise of the AO band; this is indicative of efficient energy transfer and is typical of the FRET mechanism. However, the fact that at low AO fraction a small decrease of the donor emission band provides a sharp increase of the acceptor fluorescence band indicates a very efficient energy transfer and suggests that other mechanisms besides FRET could occur. The present results are similar to the ones obtained in our previous works with Trimer A nanofibers.^16,19^ In order to assess, in more detail, the nature of the energy transfer, time-resolved ultrafast spectroscopic studies could be performed in the future. Indeed, we recently probed a very efficient coherent energy transfer^35^ occurring within DNA-guided short (10 units) chromophore stacks, between a phenanthrene antenna and a pyrene acceptor.^36^ Further work will be needed to address whether this coherent transfer can also apply to the present system. While retaining phenanthrene as the core component, our new system introduces distinct features, including a non-DNA-guided self-standing architecture, micrometer-scale SP stacks with higher-order assembly and the use of AO as the energy acceptor instead of pyrene. Eventually, we investigated the origin of the new fluorescence band developing around 575 nm, after the AO fraction has reached the value of 3.6% (*cf.*Fig. 5). For this, steady-state absorption spectra, collected at every doping fraction in the presence of SP, are shown in the region of AO low energy absorption (see Fig. S13). Interestingly, we observed a change in the shape of these spectra at a doping fraction of 3.6%. Before this fraction, AO only absorbed at 492 nm, while at higher doping fractions, the spectra revealed the growth of a shoulder at *ca.* 470 nm. In agreement with previous literature, we attribute the shoulder at 470 nm to the formation of AO aggregates, while the band at 492 nm corresponds to the absorption of the monomeric form of AO.^34^ Excitation spectra at 575 nm in the presence of SP also revealed the growth of a shoulder at 470 nm, in addition to a main excitation band at 492 nm at AO fractions higher than 3.6% (*cf.* Fig. S12). This reveals that AO aggregates are, at least partly, emissive at an emission wavelength of 575 nm. We propose that the new emission band observed at 575 nm at doping fractions higher than 3.6% (Fig. 5) corresponds in fact to the fluorescence of emissive AO aggregates. This also corroborates well with the concomitant decrease observed for the fluorescence band at 533 nm, which we propose to originate from a reduced amount of AO monomers available in the solution. This work represents, to our knowledge, one of the first examples of a light harvesting antenna, with a molecular aggregate as the energy sink. This is also interesting to notice that in the absence of SP, in otherwise identical conditions, the shoulder at 470 nm was absent from UV/visible (Fig. S16) and excitation spectra (Fig. S15), while the band at 492 nm was detected. It has been reported in the past that AO aggregation can be enhanced in the presence of negatively charged salts.^34^ We suggest that the formation of AO aggregates is induced by the interaction of AO with the negatively charged phosphate backbone of the polymer.

The present results show the ability of our newly developed hierarchical and compact system to preserve excellent light-harvesting antenna properties. Maintaining these properties while reducing the dimensionality of the SP represents an important asset for potential future integration in miniaturized devices for instance, where, likewise in a cell nucleus, space is a key parameter.

## Conclusions

In this paper, we have reported the first example of a functional synthetic SP arranging into higher order nanostructures. In the present conditions, light-harvesting nanofibers of Trimer A hierarchically organized into nanoribbons as well as large annular structures. It is noteworthy that a strong light-harvesting antenna effect was maintained for the novel structures, as evidenced by successful doping experiments, with AO as the energy acceptor. This is also the first time to our knowledge that an annular arrangement of synthetic nanofiber SP is observed. We have performed, in the present paper, the calculation of a compression factor associated with such a compact nanostructure, and we found a significant two orders of magnitude increase in value. This represents a remarkable result in synthetic SP materials science. Overall, in the present work, we came to the global conclusion that compression does not compromise optical functionality. Further potential developments of this work include deepening mechanistic studies and transfers to other systems.

## Conflicts of interest

The authors declare no conflicts of interest.

## Supplementary Material

TC-014-D6TC00644B-s001

## Data Availability

The data supporting this article have been included in the supplementary information (SI). Supplementary information: general methods, preparation of the supramolecular polymer of phenanthrene, temperature dependent steady-state fluorescence, cryo-EM, atomic force microscopy, doping experiments, and references. See DOI: https://doi.org/10.1039/d6tc00644b.
