# Supplementary material for: A hierarchical, compact and efficient phenanthrene supramolecular polymer light harvesting antenna
Source: J Mater Chem C Mater. 2026 Jun 11;14(30):13753–9. doi: 10.1039/d6tc00644b (PMC13289808; doi:10.1039/d6tc00644b)
Supplement: TC-014-D6TC00644B-s001 [file TC-014-D6TC00644B-s001.pdf]

Supplementary Information (SI) for Journal of Materials Chemistry C.
This journal is © The Royal Society of Chemistry 2026

A hierarchical, compact and efficient phenanthrene supramolecular polymer light harvesting antenna

Supporting information

Romain Brisse, Ioan Iacovache, Jovanna Jevric, Simon M. Langenegger, Benoît Zuber and Robert Häner

Table of content

General methods 3
Preparation of the supramolecular polymer of phenanthrene 4
Temperature dependent steady-state fluorescence...4
Cryo-EM 5
Complementary cryo-EM pictures..5
Size distributions of measured lengths 6
Atomic force microscopy..8
Morphology of the SP after the 1°C/min cooling ramp – Complementary picture 8
Morphology of the SP after the AO doping experiments 8
Doping experiments 9
In presence of SP 9
In absence of SP...11
Fluorescence quantum yield 12
References..13

General methods

All reagents and solvents were purchased from commercial sources and used without further purification. The water used was Milli-Q quality (ρ = 18.6 mΩ.cm^{-1}).

UV-Visible spectra were acquired with a Jasco V-730 spectrophotometer equipped with a Peltier module.

Fluorescence spectra were collected on a Jasco FP-8300 spectrophotometer, also equipped with a Peltier module, using an excitation slit of 2.5 nm and an emission slit of 5 nm. 10mm *10 mm Optical path length screw-capped quartz cuvettes (PTFE coated rubber seal) were used for either UV-Visible or Fluorescence spectroscopy. Scanning was always performed from high to low energies.

A Metrohm 913 pH Meter was used to determine pH, at 25 °C.

Samples for cryo-EM were plunge-frozen using the FEI Vitrobot Mark 4 at room temperature and 100% humidity. In brief, copper lacey carbon grids were glow discharged (air-10 mA for 20 seconds). 3 µL of the sample were pipetted on the grids and blotted for 3 seconds before plunging into liquid ethane. Sample grids were stored in liquid nitrogen. Images were acquired using a Gatan 626 cryo holder on a Falcon III equipped FEI Tecnai F20 in nanoprobe mode. The pixel size was 2.1 Å. Due to the nature of the sample, acquisition settings had to be adjusted for a low total electron dose (less than 20 e^{-}/Å^{2}) using EPU software.

Atomic Force microscopy (AFM) experiments were conducted under ambient conditions on a Nanosurf FlexAFM instrument using tapping mode. AFM samples were prepared on (3-Aminopropyl)triethoxysilane (APTES) - modified mica sheets (Glimmer “V1”, 20 mm x 20 mm, G250-7, Plano GmbH). APTES (30 µL) was pipetted into an Eppendorf tube cap and DIPEA (10 µL) was added into a second cap. Both caps were placed at the bottom of a desiccator. The freshly cleaved mica sheets were fixed at the top of the desiccator (3L). The desiccator was purged with argon and then closed. The mica sheets were left for 4 hours in the desiccator. A sample solution (15 µL) was pipetted onto an APTES-modified mica sheet. After an adsorption time of 8 min, the mica sheet was carefully rinsed with 2*1mL of Milli-Q water. Eventually, a gentle stream of argon was applied to dry the sample.

Preparation of the supramolecular polymer of phenanthrene

A stock solution of Trimer **A** was prepared in ethanol at a concentration of 14.1 µM.[1] The supramolecular polymer (SP) was always prepared as follow: 2425 µL of Milli-Q water were introduced into a quartz cuvette. Then, 65 µL of a 4.8 M Triethanolamine aqueous solution, 75 µL of 3.0 M HCl aqueous solution and 90 µL of the Trimer **A** stock solution were added to the cuvette. The final concentration of TEOA, HCl, Trimer **A** were respectively 0.12 M, 85 mM, 0.5 µM. The pH was determined to be 7.4 at 25°C.

The cuvette was heated at 85°C in the spectrophotometer. It was kept at this temperature for one hour. Then, a cooling rate of 1°C/min was applied, until the temperature of 20°C was reached. The structures were aged overnight.

Temperature dependent steady-state fluorescence

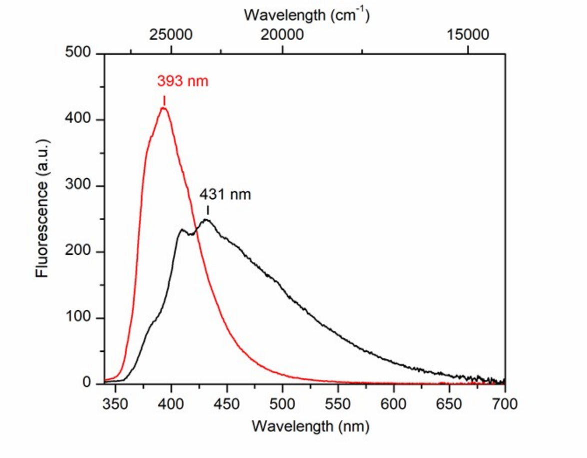

*Figure S1 – Steady state fluorescence spectra of the disassembled Trimer **A** (85°C, red) and the assembled SP (20°C, black). Conditions: TEOA (0.12 M), HCl (85 mM), Trimer **A** (0.5 µM); pH 7.4; λ_{ex}:322 nm.*

Cryo-EM

Complementary cryo-EM pictures

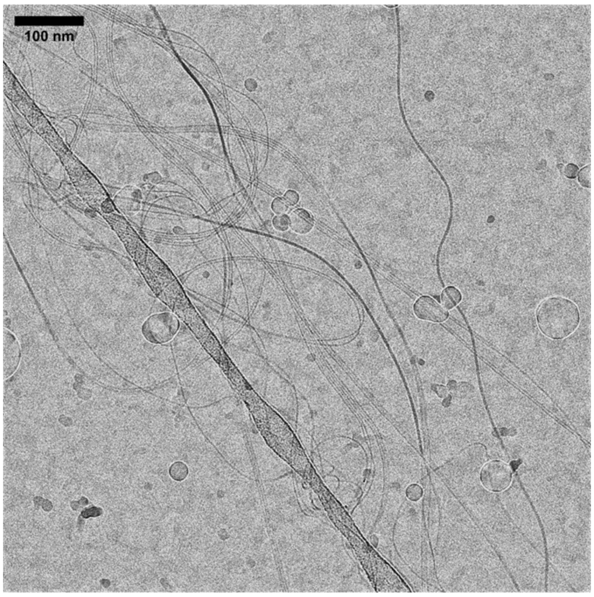

*Figure S2 – Complementary cryo-EM picture of Trimer **A** solution after self-assembly. Conditions: TEOA (0.12 M), HCl (85 mM), Trimer **A** (0.5 µM); pH 7.4.*

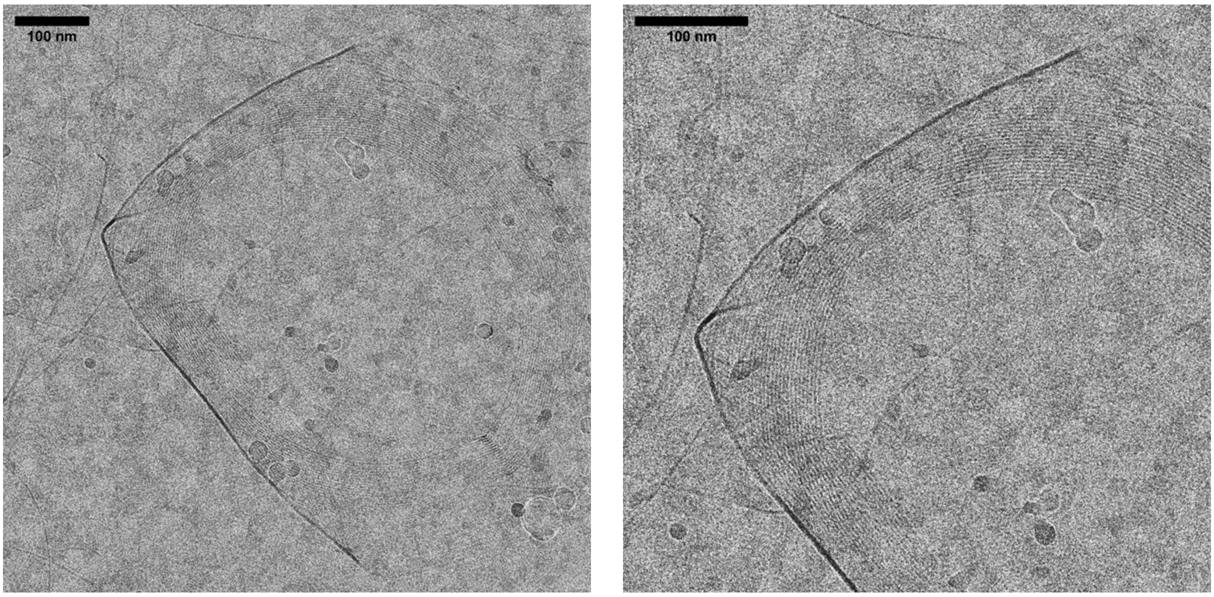

*Figure S3 – Complementary cryo-EM pictures of Trimer **A** solution after self-assembly. Left: lower magnitude; Right: higher magnitude. Conditions: TEOA (0.12 M), HCl (85 mM), Trimer **A** (0.5 µM); pH 7.4.*

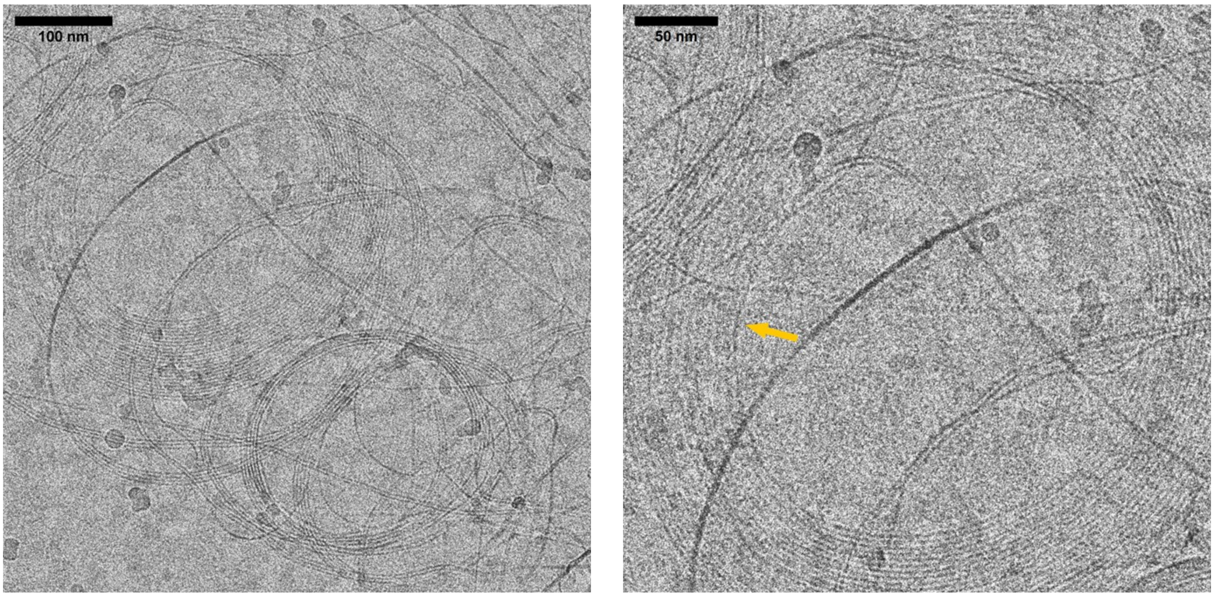

*Figure S4 – Complementary cryo-EM pictures of Trimer **A** solution after self-assembly. Left: lower magnitude; Right: higher magnitude; the orange arrow indicates a zone where nanoribbons, flipped on the edge-on orientation associate together to form another higher order nanoribbon. Conditions: TEOA (0.12 M), HCl (85 mM), Trimer A (0.5 µM); pH 7.4.*

Size distributions of measured lengths

The size distribution displayed here are obtained from cryo-EM pictures measurements.

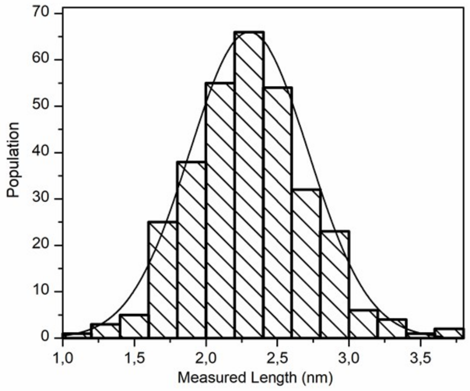

Figure S5 – Size distribution of individual fibers widths. Fibers not associated as a hierarchical structure. Total population: 315; Average size: 2.3 ± 0.4 nm.

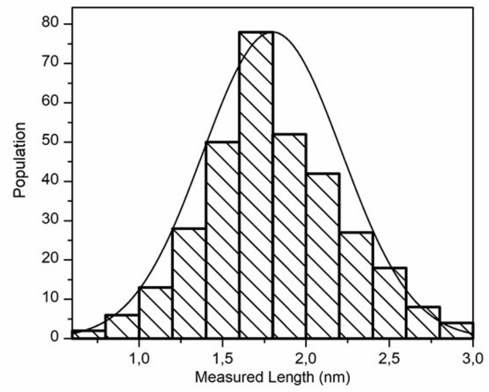

Figure S6 – Size distribution of individual fibers widths. Fibers associated as a nanoribbon (appearing in a face-on orientation). Total population: 328; Average size: 1.8 ± 0.4 nm.

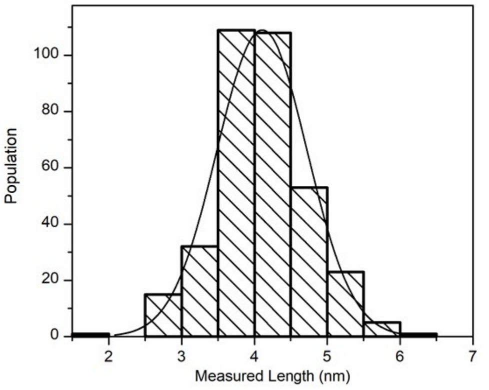

Figure S7 – Size distribution of nanoribbons widths. Ribbons appearing in an edge-on orientation. Total population: 347; Average size: 4.1 ± 0.6 nm.

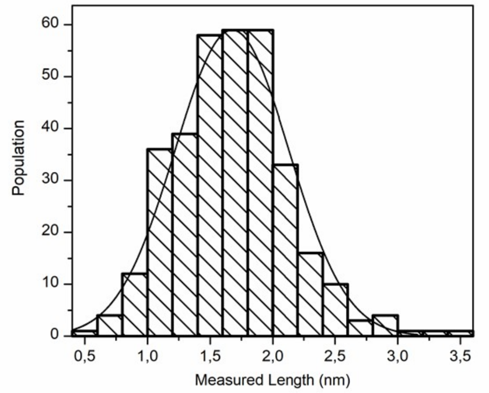

Figure S8 – Size distribution of individual fibers widths. Fibers associated as an annular structure. Total population: 337; Average size: 1.7 ± 0.5 nm.

Atomic force microscopy

Morphology of the SP after the 1°C/min cooling ramp – Complementary picture

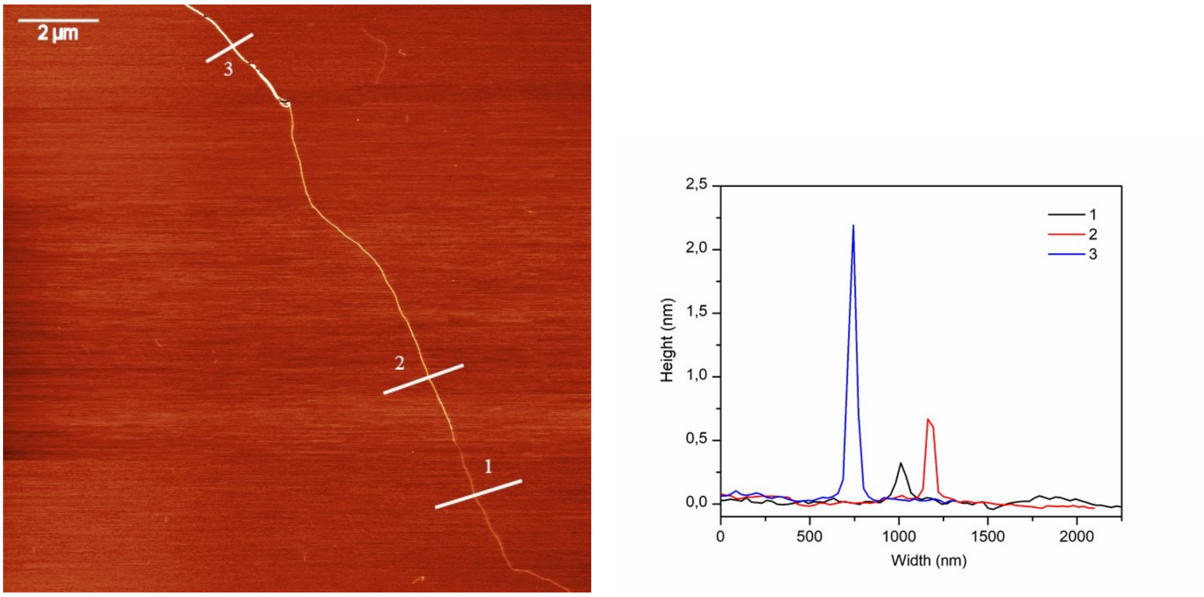

*Figure S9 – Complementary AFM picture of Trimer **A** solution after self-assembly, with the corresponding height profiles. Conditions: TEOA (0.12 M), HCl (85 mM), Trimer **A** (0.5 µM); pH 7.4. APTES-modified mica.*

Morphology of the SP after the AO doping experiments

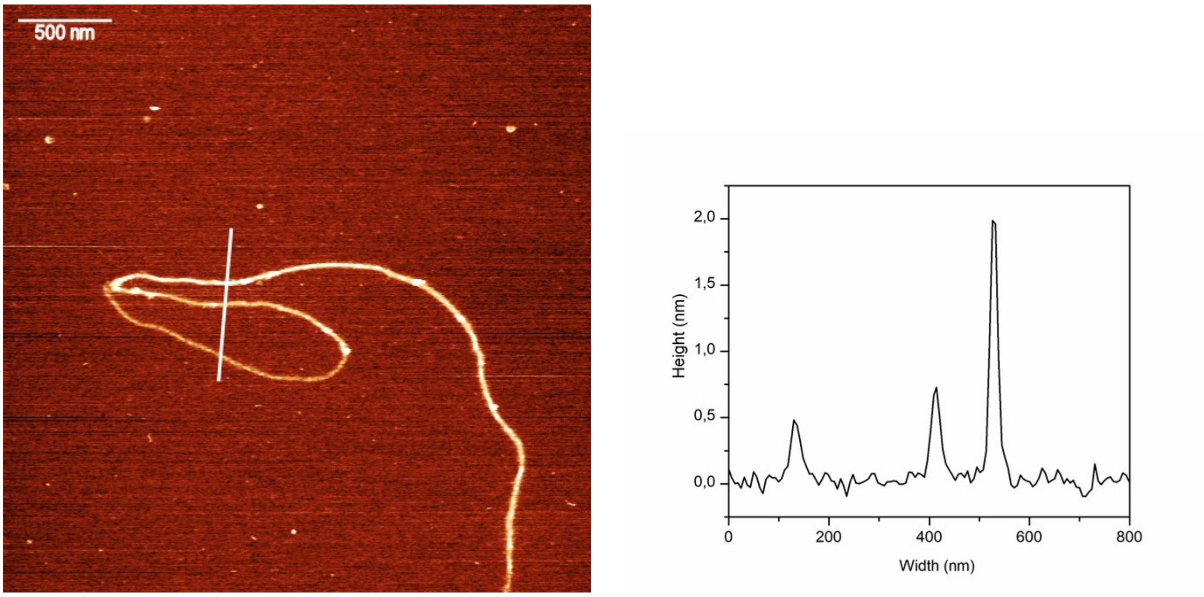

*Figure S10 – AFM picture of Trimer **A** solution after the doping experiment, with the corresponding height profile. Conditions: TEOA (0.12 M), HCl (85 mM), Trimer **A** (0.5 µM), AO (0.21 µM); pH 7.4. APTES-modified mica.*

Doping experiments

During the doping experiments, appropriate aliquots of acridine orange (AO) in ethanol were progressively added to the cuvette, in presence or in absence of SP. The AO doping fraction is referred to as the molar ratio of AO per unit of phenanthrene (one molecule of Trimer **A** contains 3 units of phenanthrene).

In presence of SP

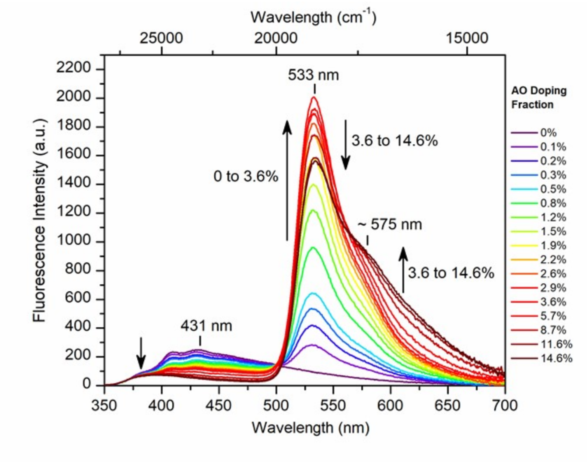

*Figure S11 – Fluorescence emission of assembled Trimer **A**, in presence of gradual AO doping fractions, at 20°C. Conditions: TEOA (0.12 M), HCl (85 mM), Trimer **A** (0.5 µM), AO doping fractions detailed in the chart; pH 7.4; λ_{ex}: 322 nm.*

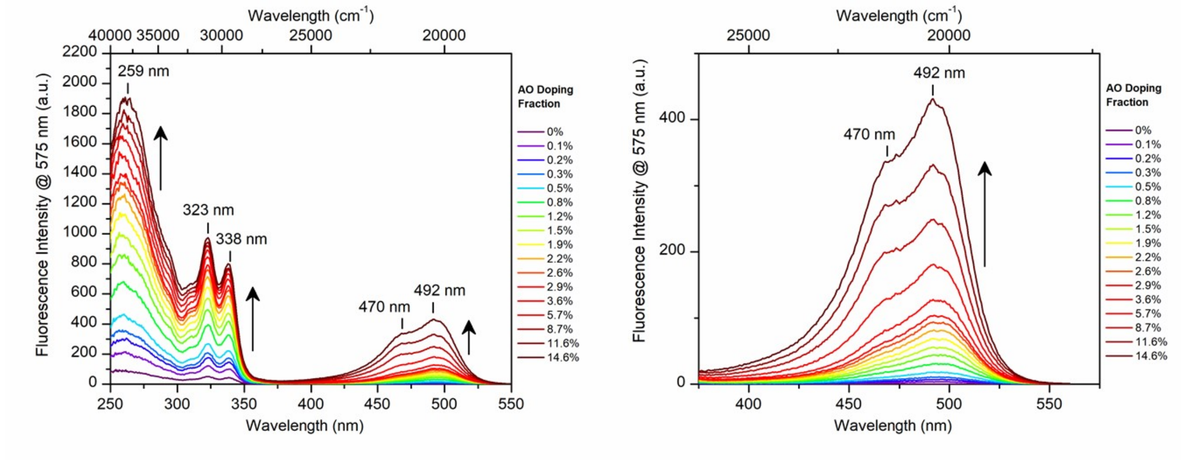

*Figure S12 – Fluorescence excitation spectra of assembled Trimer **A**, in presence of gradual AO doping fractions, at 20°C. Left: full spectrum; Right: zoom, in the low energy region. Conditions: TEOA (0.12 M), HCl (85 mM), Trimer **A** (0.5 µM), AO doping fractions detailed in the chart; pH 7.4; λ_{probe}: 575 nm.*

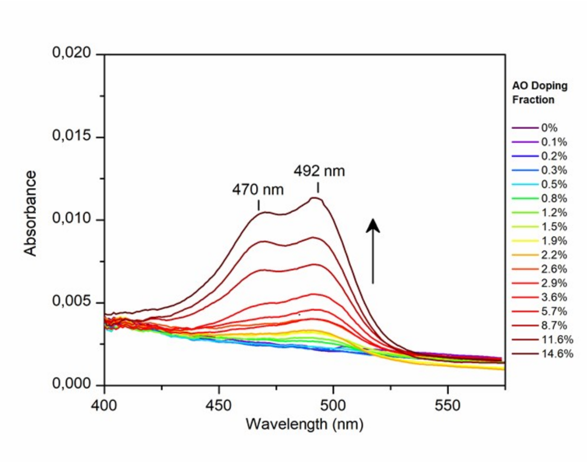

*Figure S13 – Absorption spectrum of the system, in presence of assembled Trimer **A**, at gradually increasing AO doping fractions, at 20°C. Conditions: TEOA (0.12 M), HCl (85 mM), Trimer **A** (0.5 µM), AO doping fractions detailed in the chart; pH 7.4.*

In absence of SP

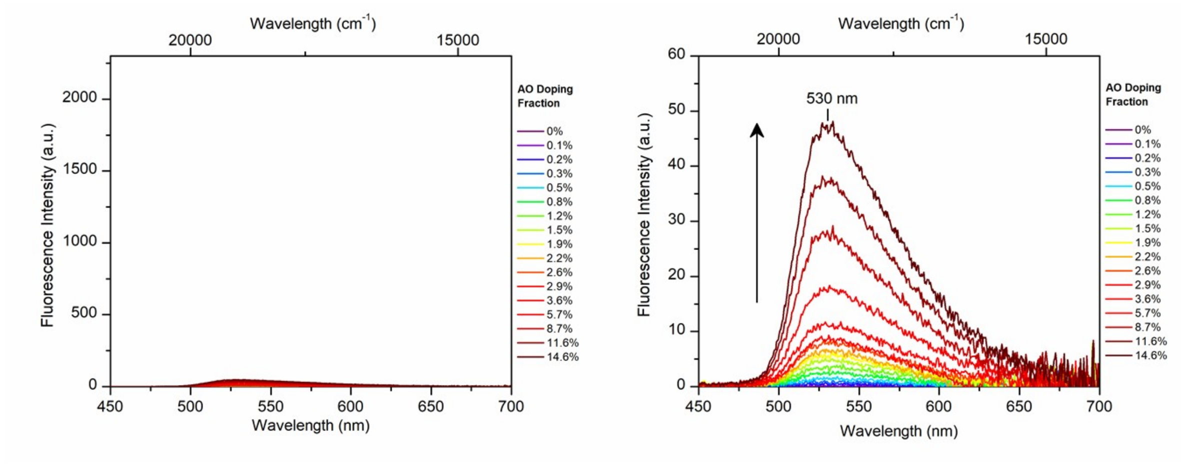

*Figure S14 – Fluorescence emission of the system, in absence of Trimer **A**, at gradually increasing AO doping fractions, at 20°C. Left: full spectrum; right: zoom in the high energy region. Conditions: TEOA (0.12 M), HCl (85 mM), Trimer **A** (0.5 µM), AO doping fractions detailed in the chart; pH 7.4; λ_{ex}: 322 nm.*

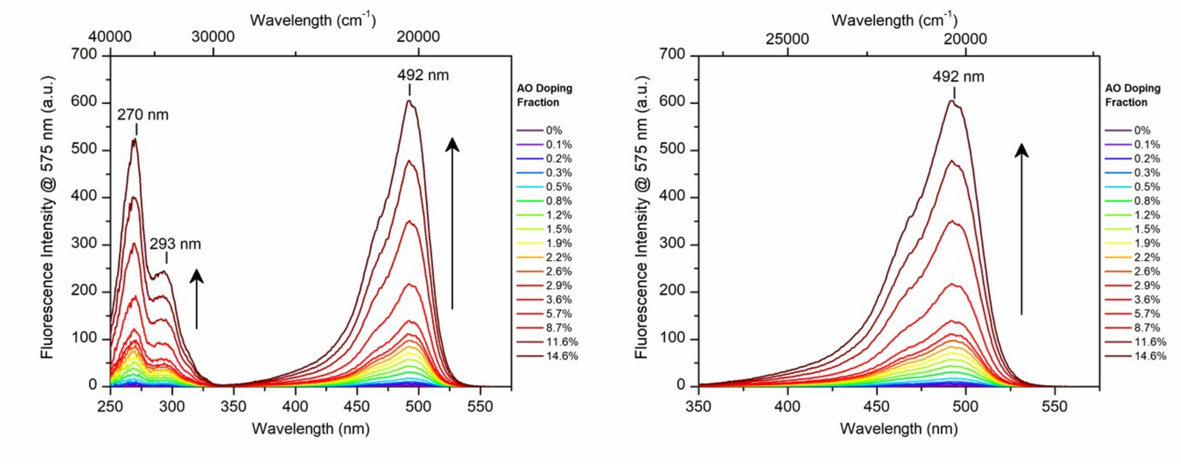

*Figure S15 – Fluorescence excitation spectra of the system, in absence of Trimer **A**, at gradually increasing AO doping fractions, at 20°C. Left: full spectrum; Right: zoom on the low energy region. Conditions: TEOA (0.12 M), HCl (85 mM), Trimer **A** (0.5 µM), AO doping fractions detailed in the chart; pH 7.4; λ_{probe}: 575 nm.*

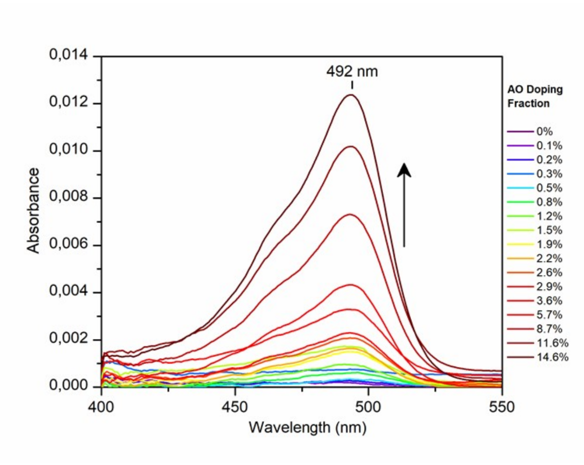

*Figure S16 – Absorption spectrum of the system, in absence of assembled Trimer **A**, at gradually increasing AO doping fractions, at 20°C. Conditions: TEOA (0.12 M), HCl (85 mM), Trimer **A** (0.5 µM), AO doping fractions detailed in the chart; pH 7.4.*

Fluorescence quantum yield

The fluorescence quantum yield was calculated according to a previously described method with quinine sulfate as the reference compound (quantum yield of quinine sulfate $\Phi_{QS} = 0.546$).[1]

Table S1 – Calculation data for the fluorescence quantum yield (Φ_F) of the SP system at gradually increasing AO doping fractions, at 322 nm. For Φ_F calculation, quinine sulfate in 0.1M in H_2SO_4 was used as a reference.

[AO] : [Phen-unit] (%)	Absorbance @ 322 nm	Integrated Fluorescence ($*10^{-3}$)	Φ_F (%)
0.0	0.036	1575	3.1
0.1	0.036	1857	3.7
0.2	0.036	2019	4.0
0.3	0.036	2198	4.4
0.5	0.037	2313	4.5
0.8	0.037	2853	5.5
1.2	0.037	3305	6.4
1.5	0.038	3601	6.8
1.9	0.039	3933	7.2
2.2	0.039	4198	7.7
2.6	0.038	4347	8.2
2.9	0.037	4487	8.7
3.6	0.037	4721	9.1
5.7	0.036	4709	9.4
8.7	0.037	4614	8.9
11.6	0.037	4490	8.7
14.6	0.037	4465	8.7

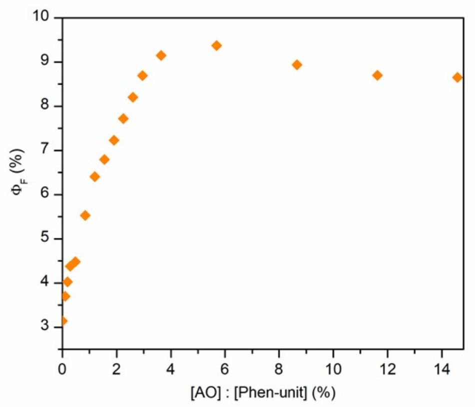

*Figure S17 – Fluorescence quantum yield of the system (Φ_F), as a function of the AO doping fraction. Conditions: TEOA (0.12 M), HCl (85 mM), Trimer **A** (0.5 µM); pH 7.4; λ_{ex}: 322 nm.*

References

1. Winiger, C. B., Li, S., Kumar, G. R., Langenegger, S. M. & Häner, R. Long-Distance Electronic Energy Transfer in Light-Harvesting Supramolecular Polymers. *Angew. Chem. Int. Ed.* **53**, 13609–13613 (2014).
